# Deep Intraspecific Divergence in the Endemic Herb *Lancea tibetica* (Mazaceae) Distributed Over the Qinghai-Tibetan Plateau

**DOI:** 10.3389/fgene.2018.00492

**Published:** 2018-10-25

**Authors:** Mingze Xia, Zunzhe Tian, Faqi Zhang, Gulzar Khan, Qingbo Gao, Rui Xing, Yu Zhang, Jingya Yu, Shilong Chen

**Affiliations:** ^1^Key Laboratory of Adaptation and Evolution of Plateau Biota, Northwest Institute of Plateau Biology, Chinese Academy of Sciences, Xining, China; ^2^College of Life Science, University of Chinese Academy of Sciences, Beijing, China; ^3^Qinghai Provincial Key Laboratory of Crop Molecular Breeding, Xining, China

**Keywords:** divergence, *Lancea tibetica*, genetic structure, phylogeography, demography, Qinghai-Tibetan Plateau

## Abstract

Qinghai-Tibetan Plateau (QTP) is an important biodiversity hub, which is very sensitive to climate change. Here in this study, we investigated genetic diversity and past population dynamics of *Lancea tibetica* (Mazaceae), an endemic herb to QTP and adjacent highlands. We sequenced chloroplast and nuclear ribosomal DNA fragments for 429 individuals, collected from 29 localities, covering their major distribution range at the QTP. A total of 19 chloroplast haplotypes and 13 nuclear genotypes in two well-differentiated lineages, corresponding to populations into two groups isolated by Tanggula and Bayangela Mountains. Meanwhile, significant phylogeographical structure was detected among sampling range of *L. tibetica*, and 61.50% of genetic variations was partitioned between groups. Gene flow across the whole region appears to be restricted by high mountains, suggesting a significant role of geography in the genetic differences between the two groups. Divergence time between the two lineages dated to 8.63 million years ago, which corresponded to the uplifting of QTP during the late Miocene and Pliocene. Ecological differences were found between both the lineages represent species-specific characteristics, sufficient to keep the lineages separated to a high degree. The simulated distribution from the last interglacial period to the current period showed that the distribution of *L. tibetica* experienced shrinkage and expansion. Climate changes during the Pleistocene glacial-interglacial cycles had a dramatic effect on *L. tibetica* distribution ranges. Multiple refugia of *L. tibetica* might have remained during the species history, to south of the Tanggula and north of Bayangela Mountains, both appeared as topological barrier and contributed to restricting gene flow between the two lineages. Together, geographic isolation and climatic factors have played a fundamental role in promoting diversification and evolution of *L. tibetica*.

## Introduction

The Qinghai-Tibetan Plateau (QTP) is one of the largest and youngest plateaus in the world, formed by several uplift events after the collision of the Indian tectonic plate with the Asian plate, about 40 Ma (million years ago) (Guo et al., 2002; Spicer et al., 2003). Further significant uplift of the QTP occurred during the periods of the East Asian summer and winter monsoons about 15 Ma (Wan et al., 2007), 10–8 Ma (Molnar et al., 1993; Harrison et al., 1995) and 3.6–2.6 Ma (Zhisheng et al., 2001). The monsoon system interacted with the glacial-interglacial cycle and produced a more variable monsoon climate during the Pleistocene (Zhisheng et al., 2001). In recent decades, considerable disagreement has arisen on the relation between the uplifting of the plateau and the East Asian monsoons. Some scholars suggest that the uplift of the QTP modified the global and East Asian climate dramatically (Bloemendal, 1989; Zhisheng et al., 2001) and triggered and intensified the Asian monsoon, which in turn strongly influenced biological processes in the region (Li and Fang, 1999). In contrast, other scholars, such as Renner (2016) and Spicer (2017), suggest that there was no obvious impact on the East Asian monsoon from the uplifting of the QTP, even they hold the opinion that uplift having reached average heights of 4–5 km since the mid-Eocene. However, these climatic oscillations did affect the demography of some species, leading to their range shifting or extinction. Furthermore, the harsh climate of this region may have improved the adaptability of some local organisms (Davis and Shaw, 2001; Hewitt, 2004; Wan et al., 2016).

Numerous endemic species occur in the QTP and adjacent highlands, which represent centers of the preservation of ancient species and the differentiation of young species (Wu, 1988; Myers et al., 2000; Liu et al., 2012). A popular but rarely proved hypothesis is that the uplift of mountains creates environmental conditions (such as dispersed barriers or new habitats) that increase the rate of speciation (Xing and Ree, 2017). However, several phylogeographic studies have shown that certain species may have retreated during the ice age to refugia located at edge of the QTP, and recolonized the QTP and the adjacent highlands after the ice age, e.g., *Juniperus przewalskii* (Zhang et al., 2005), *Metagentiana striata* (Chen et al., 2008), and *Pedicularis longiflora* (Yang et al., 2008). Recent studies on *Aconitum gymnandrum* (Wang et al., 2009), *Hippophae rhamnoides* (Jia et al., 2012), and *Spiraea alpina* (Gulzar et al., 2018) suggest that some species also survived in the QTP at high altitude during the glacial period. For those species, multiple refugia may have remained during the glacial period, some on the QTP and others on its edge (Liu et al., 2012). For every species that has been researched, there is a species-specific feature in their evolutionary histories, even in some closely related species, such as *S. alpina* and *S. mongolica* (Gulzar et al., 2018). Therefore, further phylogeographic studies of a wider range of species are necessary to improve and refine the model for differentiation and formation of species in the region.

According to the present taxonomical treatment, *Lancea* Hook. f. et Thoms. is a small genus of the Mazaceae with only two species, *L. tibetica* and *L. hirsuta* (Hong et al., 1998). As a traditional Tibetan medicinal plant, *L. tibetica* has been used in the treatment of leukemia, intestinal angina, heart disease, and cough (Song et al., 2011b). Phytochemical studies on *L. tibetica* suggest that it contains more than 71 compounds that have pharmacodynamic effects, including anti-tumor, antioxidant, and hypoglycemic-inhibiting activities (Song et al., 2011a; Liu et al., 2014, 2015). *L. tibetica* is a perennial species endemic to the QTP, widely distributed in alpine meadows at altitudes of 2,000–4,500 m (Hong et al., 1998). The generation time for *L. tibetica* is 2 years according to our preliminary field observations. Under the inferior living condition, local human harvest the wild populations puts extra pressure on *L. tibetica* threatened with extinction (Tian et al., 2016). In this study, by combining ecological niche modeling and molecular data, we investigated the historical, genealogical and promoted diversity of *L. tibetica*, to gain insights into its intraspecific divergence and spatiotemporal population dynamics. Our study provides an important advance in knowledge of the population dynamics of endemic species on the QTP.

## Materials and Methods

### Population Sampling and Experimental Protocols

**FIGURE 1:**
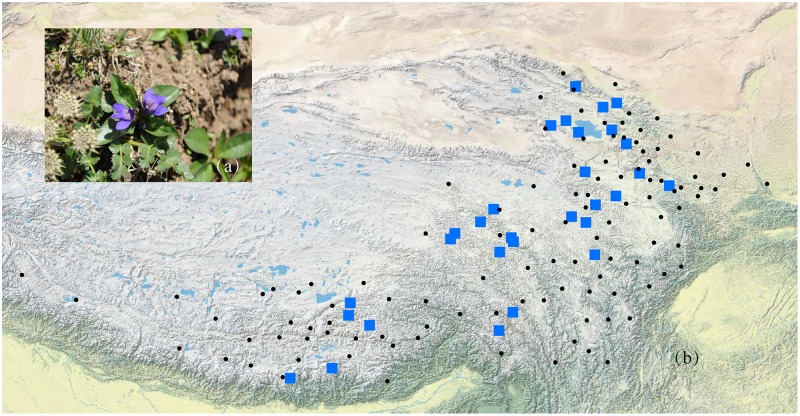
A photograph of *L. tibetica* plant **(a)** and a map of sampling coverage in this study **(b)**. Black dots represent herbarium records in CVH, and blue squares represent sampled populations in this study.

According to the Flora of China (Hong et al., 1998) and herbarium records from the Chinese Virtual Herbarium (CVH ^1^), *L. tibetica* mainly occurs in Gansu, Qinghai, Sichuan, Xizang, and Yunnan in China. It should be noted that few CVH herbarium records of *L. tibetica* are mainly before the 1960s. Some locations with just one or two records are difficult to sample again in our field investigation. In this study, a total of 429 individuals were collected from 29 populations covering the major distribution range of *L. tibetica* (Table 1 and Figure 1). Fresh leaves were sampled, dried *in silica* gel and kept at -20°C until DNA extraction. *Pedicularis rhinanthoides* and *P. chinensis* were also sampled as outgroups (sample information may be found under the GenBank accession numbers MH628332–MH628339). All the sampling locations were geo-referenced, and voucher specimens deposited into the Herbarium of Northwest Plateau Institute of Biology (HNWP), Chinese Academy of Sciences.

**Table 1:** Sample locations, sample size and haplotype frequencies for 29 populations of *L. tibetica.*

P.	Location	Latitude (N)	Longitude (E)	Altitude (m)	Plastid haplotype	Genotype	*H*_d_	*P*_i_ (100×)
1	Yadong, XZ	27°47′	89°08′	4350	A(6), F(1)	G2(7)	0.28571	0.013
2	Luozha, XZ	28°08′	90°41′	4566	A(7), B(1)	G1(1), G2(3), G3(3), G4(1)	0.25000	0.012
3	Milashan, XZ	29°42′	92°03′	4136	A(20)	G2(20)	0	0
4	Linzhi, XZ	30°04′	91°16′	4232	A(7), R(4), S(1)	G2(11), G3(1)	0.59091	0.030
5	Dangxiong, XZ	30°32′	91°20′	4381	A(8)	G2(3), G4(4)	0	0
6	Basu, XZ	29°31′	96°46′	4140	A(3), D(1), E(22)	G2(1), G4(4), G8(12), G9(2), G10(8)	0.28000	0.077
7	Jieduo, QH	32°52′	95°00′	4327	A(12), D(3), K(1)	G5(3), G7(4), G8(12)	0.42500	0.071
8	Zaqing, QH	33°5′	95°9′	4289	D(17)	G5(7), G7(2), G8(10)	0	0
9	Xialaxiu, QH	32°23′	96°47′	3770	A(7), D(2), K(6), M(1)	G2(3), G7(5), G8(8)	0.69167	0.093
10	Batang, QH	32°46′	97°18′	4100	A(10), D(5), I(3)	G7(4), G8(14)	0.62092	0.092
11	Yushu, QH	32°55′	97°13′	3667	D(14), I(2), J(1)	G7(4), G8(13)	0.32353	0.055
12	Zhiduo, QH	33°29′	96°05′	4370	A(9), D(3), I(2), K(4), L(1)	G5(4), G7(8), G8(7)	0.73099	0.098
13	Qumalai, QH	33°58′	96°34′	4570	D(3), F(1), I(7), K(7)	G5(4), G7(9), G8(5)	0.70588	0.127
14	Seda, SC	32°17′	100°16′	3926	A(4), D(2), N(4), Q(1)	G6(1), G7(5), G8(6)	0.76364	0.094
15	Dari, QH	33°41′	99°26′	4028	D(3), M(1), N(3)	G7(2), G8(2), G9(2), G11(1)	0.71429	0.039
16	Dawu, QH	33°28′	99°56′	3872	D(28)	G5(13), G6(5), G7(7), G8(2), G13(1)	0	0
17	Gande, QH	34°07′	100°18′	4020	D(13), N(1), P(2)	G5(3), G7(7), G8(5), G13(1)	0.34167	0
18	Henan, QH	34°27′	101°02′	3657	D(10)	G5(5), G7(1), G8(2), G9(2)	0	0
19	Xinghai, QH	35°20′	99°54′	3622	C(2), D(10)	G5(7), G6(2), G7(3)	0.30303	0.014
20	Tongren, QH	35°16′	101°54′	3036	C(1), D(8)	G5(1), G7(7), G8(1)	0.22222	0.010
21	Hezuo, GS	34°50′	103°00′	3220	A(1), C(1), D(8)	G5(1), G7(1), G8(7), G9(2)	0.37778	0.044
22	Guide, QH	36°21′	101°26′	3782	A(1), D(12)	G5(5), G7(6), G8(2)	0.15385	0.028
23	Xihai, QH	36°52′	100°54′	3137	A(3), D(9), H(1)	G5(2), G6(1), G7(3), G8(4), G12(3)	0.50000	0.078
24	Gonghe, QH	36°46′	99°40′	3396	D(9), N(2), O(1)	G5(7), G9(5)	0.43939	0.023
25	Dulan, QH	37°01′	98°39′	3445	D(6)	G7(3), G8(2), G13(1)	0	0
26	Tinajun, QH	37°11′	99°13′	3340	D(13), G(1)	G5(2), G6(1), G7(4), G8(4), G9(2), G11(1)	0.14286	0.007
27	Gangcha, QH	37°42′	100°34′	3442	D(14), H(2)	G5(4), G7(11), G8(1)	0.23300	0.011
28	Menyuan, QH	37°51′	101°04′	3636	D(31)	G5(10), G6(1), G7(17), G8(2), G11(1)	0	0
29	Qilian, QH	38°26′	99°33′	3296	D(18), H(1)	G5(7), G6(5), G7(6), G8(1)	0.10526	0.005
Total							0.62470	0.108

*P., population code; XZ, Xizang Autonomous Region; QH, Qinghai Province; GS, Gansu Province; SC, Sichuan Province; H_d_, haplotype diversity; P_i_, nucleotide diversity.*

Genomic DNA was extracted from approximately 20 mg of dried leaves using a modified cetyltrimethylammonium bromide (CTAB) method (Doyle, 1987). Four intergenic spacers of chloroplast DNA (cpDNA) (genes *trn*H-*psb*A, *mat*K, *trn*L-F and *rbc*L) and two nuclear ribosomal internal transcribed spacer regions (ITS1 and ITS2) were amplified for all samples (White et al., 1990; Baldwin, 1992; Olmstead and Michaels, 1992; Sang et al., 1997). PCR amplification was performed using the following protocol: 25-μL reaction mixtures containing 30–50-ng genomic DNA, 2.5 μL of 10× PCR buffer (containing Mg^2+^), 1 μL of dNTPs (each 10 mM), 0.5 μL of each primer (50 μM), and 1 U of Taq DNA polymerase (Takara, China). The amplification temperature followed a profile of 95°C for 1 min; 30 cycles of 95°C for 30 s, 55°C for 30 s and 72°C for 1 min 30 s; extension at 72°C for 10 min. PCR products were sequenced with an ABI 377XL DNA sequencer (Applied Biosystems). The program CLUSTAL X (Thompson et al., 1997) was used to perform alignment of all the sequences and the alignment was checked manually in BioEdit v7.0.5 (Hall, 1999). All sequences have been deposited in GenBank under accession numbers MG818228–MG818245, MH605185–MH605197, and MH628332–MH628339.

### Genetic Variation and Population Genetic Structure

During all analyses, insertion–deletion polymorphisms (indels) were coded as presence/absence characters. After alignment, cpDNA haplotypes and ITS genotypes were identified and distinguished using DnaSP v5.0 (Librado and Rozas, 2009). The level of genetic variation, total haplotype diversity (*H*d) and nucleotide diversity (*P*i) were also calculated in DnaSP. The program PERMUT (Pons and Petit, 1996) was used to estimate within-population diversity (*H*_S_), total gene diversity (*H*_T_), genetic differentiation (*G*_ST_) and population subdivision of phylogenetically ordered alleles (*N*_ST_) (Nei, 1987; Grivet and Petit, 2002). The *G*_ST_ value represents the degree of genetic differentiation among the population and was calculated as *G*_ST_= (*H*_T_ -*H*_S_)/*H*_T_ (Raymond and Rousset, 1995). The U-statistical method was used to compare *G*_ST_ and *N*_ST_ (using 1,000 repeat replacement tests) and to check the geographical distribution pattern.

Population subdivision analysis was performed in the program SAMOVA v1.0 (Dupanloup et al., 2010), to define groups that are geographically homogeneous and genetically differentiated. The analysis used the data from cpDNA, run for *K* = 2–10, starting from 1,000 random initial conditions for each run, to obtain the maximal value of *F*_CT_ for the most appropriate *K*-value and grouping method. Genetic differentiation based on cpDNA was estimated through analysis of molecular variance (AMOVA) as implemented in the program ARLEQUIN v3.5 (Excoffier and Lischer, 2010). To calculate the average effective gene flow, we used the formula *N*_m_ = ([1/*F*_ST_]-1)/2. Genetic distances were estimated in ARLEQUIN with 1,000 permutation tests.

### Phylogeny and Demographic History Based on cpDNA Sequences

Relationships among cpDNA haplotypes were constructed via a maximum-parsimony median-joining network using NETWORK 4.6 with default parameters (Bandelt et al., 1999). Phylogeny of cpDNA haplotypes was estimated using MrBayes 3.1.2 (Drummond et al., 2012). *P. rhinanthoides* and *P. chinensis* were selected as outgroups, as *Pedicularis* and *Lancea* were formerly in the same family Scrophulariaceae (Hong et al., 1998). The best-fitting GTR + G + I model was selected by MrModeltest 2.3 (Nylander, 2004) using the Akaike Information Criterion. For MrBayes, two independent Markov-chain Monte Carlo analyses for 100,000,000 generations were performed with a random starting tree. One cold and three heated chains were run simultaneously, with trees sampled every 1,000 generations, and discarding the first 25% as burn-in. FIGTREE 1.3.1 (Rambaut, 2009) was used to display the tree.

Tajima’s *D* and Fu’s *F*_S_ statistics were calculated to test for evidence of range expansion (Tajima, 1989; Fu, 1997; Jaeger et al., 2005). A significant value for *D* or a significantly large negative value for *F*_S_ may be the result of population expansion (ArisBrosou and Excoffier, 1996). To analyze the dynamic size of the populations, we performed mismatch distribution as implemented in ARLEQUIN. The observed and expected mismatch distribution of the sum of squared deviation (SSD) and Harpending’s raggedness index (HRI) were used as test statistics. A unimodal shape of the mismatch distribution provides evidence of sudden population expansion during the history of a species. All the tests were implemented in ARLEQUIN v3.01 (Rogers and Harpending, 1992) with 1,000 significant permutations. When the sudden expansion model was accepted, the formula τ = 2*ut* was used to estimate the age of expansion (*t*), where τ is the total number of mutations and *u* is the mutation rate per generation for the whole analyzed sequence. The value of u is calculated as *u* = μ*kg*, where μ is the substitution rate per nucleotide site in 1 year, *k* is mean sequence length of the analyzed DNA region and *g* is the generation time of the plant. We used the substitution rates (2 × 10^-9^ s s^-1^ year^-1^) of cpDNA (Wolfe et al., 1987) to estimate the expansion time of both clades.

### Divergence Time Analysis Based on ITS Sequences

There are few reports of fossil data of Lamiales, and only a few fossil records [*Fraxinus* L. (Call and Dilcher, 1992; Magallon, 2000); *Catalpa* Bur. (Meyer and Manchester, 1997)] are reliable (Manchester, 1999). Based on these fossil records, Nie et al. (2006) analyzed the divergence time of Lamiales, showed the divergence between *Mazus reptans* and *L. tibetica* was around 25 Ma. The sequence of *M. reptans* (LC027734) was used as the outgroup in analyzing ITS data (Nie et al., 2006). The GTR + I base substitution model was selected with a loose molecular clock model of the uncorrelated index in BEAST 1.5.0 (Drummond and Rambaut, 2007). The two independent models were analyzed and combined using LogCombiner v1.5.3. Convergence was traced using TRACER v1.7 (Rambaut et al., 2018). The program TreeAnnotator v1.5.3 (Rambaut et al., 2018) was used to summarize the maximum credible tree. Finally, a tree showing ages for each branch was displayed in FigTree v1.3.1 (Rambaut, 2009).

### Ecological Niche Modeling

During the field work, the locations of the sampling sites were recorded using GPS (Table 1). To infer the potential geographic range and the effects of past climatic oscillations on *L. tibetica* through one complete glacial-interglacial cycle, we performed species distribution models based on current, mid-Holocene (6 ka), last glacial maximum (20 ka), and last interglacial (135 ka) periods (Otto-Bliesner et al., 2006). We simulated the species distribution models GBM: generalized boosted models (Ridgeway, 2004); SRE: surface range envelop (Busby, 1991); GLM: generalized linear model (Mccullagh and Nelder, 1989), CTA: classification tree analysis (Breiman et al., 1984); ANN: artificial neural network (Lecun and Bengio, 1996); FDA: flexible discriminant analysis (Hastie, 1994); MARS (Friedman, 1991); RF: random forests (Breiman, 2001); and MAXENT (Phillips et al., 2004) using R package biomod2 v3.1-64 (Thuiller et al., 2014), supported with additional packages rworld map, rgdal, dismo, and SDMTools. To evaluate the effectiveness of these algorithms we used TSS and ROC values >0.7 to assemble the raster layers using median values. A total of 13 bioclimatic variables were chosen (annual mean temperature, mean diurnal range, isothermality, temperature seasonality, maximum temperature of warmest month, minimum temperature of coldest month, temperature annual range, mean temperature of wettest quarter, mean temperature of driest quarter, mean temperature of warmest quarter, annual precipitation, precipitation of wettest month, and precipitation of driest month) with low correlation and high informativeness after a jackknife procedure on the 19 bioclimatic variables downloaded from the WorldClim database (Robert et al., 2005).

We selected the maximum entropy model and machine learning algorithm as implemented in MAXENT v3.3.3k (Phillips et al., 2006; Phillips and Dudík, 2008) to predict suitable climate models for both lineages. MAXENT can produce a useful model with a small sample size (Hernandez et al., 2006; Pearson et al., 2007; Wisz et al., 2008; Anderson and Gonzalez, 2011). We used all the 19 bioclimatic variables from 1950 to 2000, downloaded from the WorldClim database (Hijmans et al., 2005). In addition, we selected ten environmental variables (annual mean temperature, mean diurnal range, isothermality, maximum temperature of warmest month, minimum temperature of coldest month, mean temperature of driest quarter, mean temperature of warmest quarter, mean temperature of the coldest quarter, precipitation seasonality, precipitation of coldest quarter) to perform the tests. The restricted dataset was used to avoid including highly correlated variables and prevent potential overfitting (Peterson and Nakazawa, 2008). Model performance was evaluated by the area under the receiver operating characteristic curve (AUC) using the program MAXENT. We used a jackknife (or ‘leave-one-out’) procedure to train and test the model. Values between 0.7 and 0.9 indicated good discrimination (Swets, 1988).

To measure the niche similarity between lineages, we used ENMTools 1.3 (Warren et al., 2008, 2010) to calculate Schoener’s *D* and Warren’s *I* indices (Warren et al., 2008) and quantify niche overlap: a value of 0 means ecological niches do not overlap at all, and 1 means the habitats are estimated to be equally suitable for both lineages. The overlap test was performed in layers using the program MAXENT. A niche identity test was obtained based on 200 pseudo-replicates to generate a distribution of the expected values of each index. The significance of observed and expected indices were estimated using SPSS v20.0 (IBM Corp, 2013).

## Results

### Sequencing, Genetic Variation, and Population Genetic Structure

**FIGURE 2:**
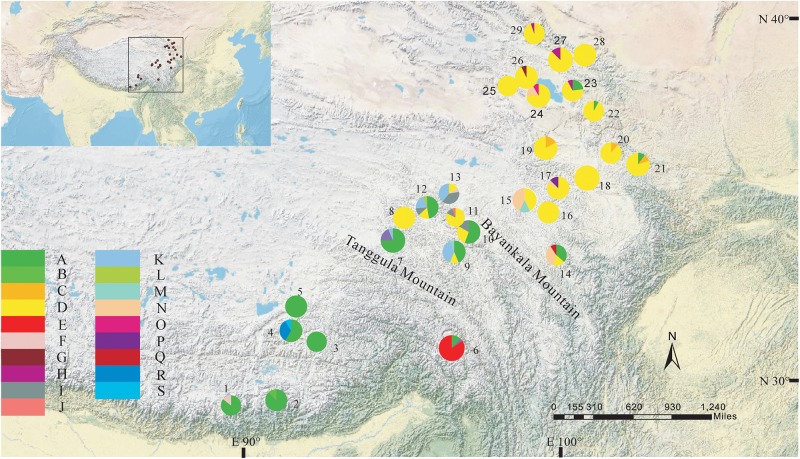
Geographic distribution of haplotypes detected from the combined cpDNA sequences of *L. tibetica* (population codes as detailed in Table 1).

The total alignment length of four chloroplast gene regions (*trn*H-*psb*A, *mat*K, *trn*L-F, and *rbc*L) in all individuals was 2,179 bp, included 14 substitutions and four indels (also coded as substitutions during analysis, Table 2). Based on those polymorphisms, we identified a total of 19 haplotypes (A–S), which were asymmetrically distributed across the 29 populations (Figure 2). The total estimated haplotype diversity (*H*d) was 0.6247 and nucleotide diversity (*P*i) was 0.00108 (Table 1). At the population level, the populations 9–15 showed a higher *H*d and *P*i. Haplotypes A and D were widely distributed in the south and north ranges, respectively (Figure 2). Populations 9, 10, and 12–15 showed higher haplotype and nucleotide diversities.

**Table 2:** Variable nucleotide sites in four chloroplast DNA fragments, allowing 19 haplotypes to be identified in *L. tibetica.*

Plastid haplotype	*mat*K			*psb*A-*trn*H						*trn*L-F			*rbc*L				
	129	416	617	42	98	258	261	264	271–276	277	120	432	581	46	205	243	322	601
A	T	C	T	G	A	T	A	–	–	A	A	T	G	G	C	T	G	C
B	T	C	T	G	A	T	A	–	–	A	T	T	G	G	C	T	G	C
C	G	C	T	G	A	T	A	–	–	A	A	C	A	G	C	T	G	C
D	G	C	T	G	–	T	A	–	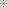	A	A	C	A	G	C	G	G	C
E	G	C	T	T	A	A	T	A	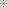	A	A	C	A	G	C	G	G	C
F	T	C	T	G	A	T	A	–	–	A	A	T	G	G	C	G	G	C
G	G	C	T	G	–	T	A	–	–	A	A	C	A	G	C	T	G	C
H	G	C	G	G	–	T	A	–	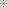	A	A	C	A	G	C	G	G	C
I	T	C	G	G	A	T	A	–	–	A	A	T	G	G	C	T	G	C
J	T	C	T	G	A	T	A	–	–	A	A	C	A	G	C	G	G	C
K	T	C	T	G	A	T	A	–	–	A	A	T	G	G	T	T	G	T
L	T	C	T	G	A	T	A	–	–	A	A	T	G	A	C	T	G	C
M	G	C	T	G	A	T	A	–	–	A	A	T	G	G	C	T	G	C
N	G	C	T	G	–	T	A	–	–	A	A	C	A	G	C	G	G	C
O	G	C	T	G	–	T	A	–	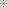	A	A	C	A	G	T	T	G	T
P	G	C	T	G	–	T	A	–	–	–	A	C	A	G	C	G	G	C
Q	G	C	T	G	A	T	A	–	–	A	A	C	A	G	C	G	G	C
R	T	C	T	G	A	T	A	–	–	A	A	T	G	G	C	T	A	C
S	T	T	T	G	A	T	A	–	–	A	A	T	G	G	C	T	G	C

*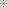, TAAAAT.*

**FIGURE 3:**
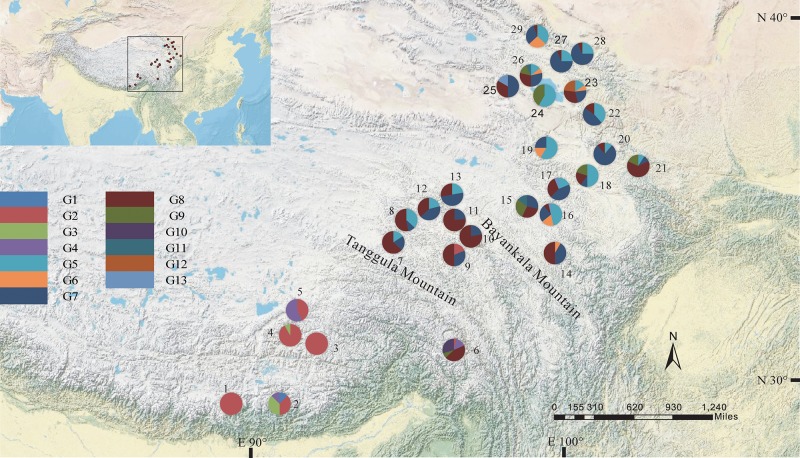
Geographic distribution of genotypes detected from the ITS sequences of *L. tibetica* (population codes as detailed in Table 1).

The total alignment length of ITS in all individuals was 693 bp, which included eight substitutions that enabled us to identify thirteen genotypes (G1–G13; Table 3). In combination with the geographical distribution of *L. tibetica*, our results indicated that the G1–G4 genotypes were mainly distributed to the south of the Tanggula Mountains, while the other genotypes were found to the north (Figure 3).

**Table 3:** Variable nucleotide sites in nuclear ribosomal internal transcribed spacer (ITS) sequences in 13 genotypes identified in *L. tibetica.*

Genotype	ITS							
	104	163	284	301	356	527	590	641
G1	G	A	A	G	G	T	C	G
G2	G	A	A	G	G	A	C	G
G3	A	A	A	G	G	A	C	G
G4	G	A	A	A	G	A	C	G
G5	G	G	G	G	G	T	C	G
G6	G	G	G	G	A	A	C	G
G7	G	G	G	G	G	A	C	G
G8	G	A	G	G	G	A	C	G
G9	G	G	G	G	G	T	A	G
G10	G	A	G	G	G	A	C	A
G11	G	G	G	G	G	A	A	G
G12	G	A	G	G	G	A	A	G
G13	G	A	G	G	G	T	C	G

The program SAMOVA divided all the populations into two groups based on the chloroplast sequences, corresponding to the south and north lineages. The south lineage included populations 1–7, 9, 10, 12, and 13 while the north lineage included populations 8, 11, and 14–29, although the *F_CT_* was not the highest. The *F_CT_* values changed very little with increasing number of groups (*K*) and were highest at *K* = 5 when populations 6, 14, and 15 formed three groups (these populations had a high proportion of private haplotypes). The average genetic diversity (*H*_S_) was 0.311, while the total genetic diversity (*H*_T_) was 0.644. The *N*_ST_ (0.662) was significantly higher than *G*_ST_ (0.507), as shown by a *U*-test (*P* < 0.01), indicating significant phylogeographical structure in *L. tibetica*. AMOVA revealed that 61.50% of genetic variation was partitioned among groups, 15.50% among populations within the group, and 22.94% within populations (Table 4). Moreover, the average gene flow among the populations and between the two groups of *L. tibetica* was 0.249 and 0.149, respectively.

**Table 4:** AMOVA for cpDNA data among two clades and all populations of *L. tibetica.*

Source of variation	d.f.	Sum of squares	VC	PV (%)	Fixation
Total populations					
Among populations	28	836.702	1.96575	66.76	*F*_ST_ = 0.66758^∗^
Within populations	400	391.54	0.97885	33.242	
Total		1228.242			
North clade vs. south clade					
Among groups	1	549.127	2.62536	61.54	*F*_ST_ = 0.77057^∗^
Among populations within groups	27	287.576	0.66219	15.52	*F*_SC_ = 0.40352^∗^
Within populations	400	391.540	0.97885	22.94	*F*_CT_ = 0.61536^∗^

*d.f., degrees of freedom; VC, variation component; PV, percentage of variation; F_ST_, correlation within populations relative to total; F_CT_, correlation of haplotypes within groups relative to total; F_SC_, correlation within populations relative to groups; ^∗^ represents P < 0.01, with 1,000 permutations.*

### Phylogeny and Demographic History Based on cpDNA Sequences

**FIGURE 4:**
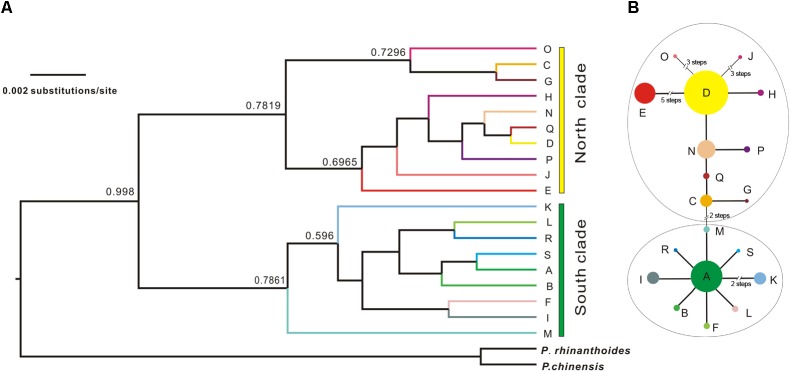
Bayesian tree of *L. tibetica* with *P. rhinanthoides* and *P. chinensis* as an outgroup, based on analysis of cpDNA sequences. **(A)** Bayesian tree of 19 *L. tibetica* lineages and two *Pedicularis* species: the numbers at the branches are posterior probability values. **(B)** maximum-parsimony median-joining network of the genealogical relationship among the 19 cpDNA haplotypes. Each circle denotes a single haplotype, shown with the area in proportion to its frequency. The numbers near the slashes across network branches indicate the number of mutational steps. The remaining branches represent single mutational steps.

The Bayesian inference tree topology of the 19 cpDNA haplotypes strongly supported the hypothesis of two lineages (south and north; Figure 4A). Haplotypes in the south lineage occurred in populations from the south of the QTP, and haplotypes in the north lineage occurred in populations from the north. The maximum-parsimony median-joining network also grouped all the cpDNA haplotypes into two major groups (south and north) separated by two mutational steps (Figure 4B).

**FIGURE 5:**
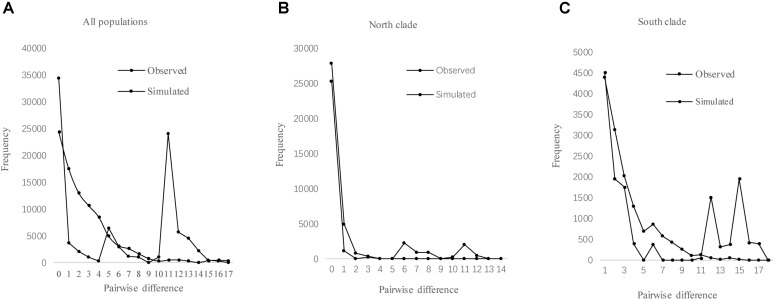
Mismatch distribution analysis for cpDNA sequence data of all populations **(A)**, north clade **(B)**, and south clade **(C)** in *L. tibetica*.

The results of Tajima’s *D* and Fu’s *F*S was not significant. However, the observed mismatch distributions of haplotypes for each lineage failed to reject the spatial expansion model (*SSD, H*_Rag_ values *P* > 0.05; Table 5 and Figure 5). The observed mismatch distributions of the whole population rejected the spatial expansion model (*SSD, H*_Rag_ values *P* < 0.05; Table 5). Based on the range of the plastid DNA substitution rate, a haplotype sequence length of 2,179 bp and 2-year generation time, the expansion of the south lineage was estimated to have occurred at 0.727 Ma (with a confidence interval 0.024–1.412 Ma), and that of the north at 0.172 Ma (with a confidence interval 0.021–0.201 Ma).

**Table 5:** Mismatch distribution analysis and neutrality tests for pooled populations of lineages.

Group	*SSD* (*P*-value)	*H*_Rag_ (*P*-value)	Tajima’s *D* (*P*-value)	Fu’s Fs (*P-*value)	Parameter (τ)	Expansion time (*t*)
All populations	0.14(0.02)	0.224(0.05)	0.265(0.68)	2.873(0.81)	NC	NC
North clade	0.03(0.10)	0.517(0.62)	-1.24(0.10)	-1.214(0.36)	3.000(0.361–3.500)	0.172(0.021–0.201) Ma
South clade	0.05(0.46)	0.089(0.57)	0.30(0.68)	5.06(0.97)	12.675(0.420–24.616)	0.727(0.024–1.412) Ma

*Estimates were acquired under a model of spatial expansion using ARLEQUIN. τ, time in several generations elapsed since the sudden expansion episode; HRag, Harpending’s raggedness index; SSD, the sum of squared deviations; NC, not calculated; Ma, million years ago.*

### Divergence Time Analysis Based on ITS Sequences

**FIGURE 6:**
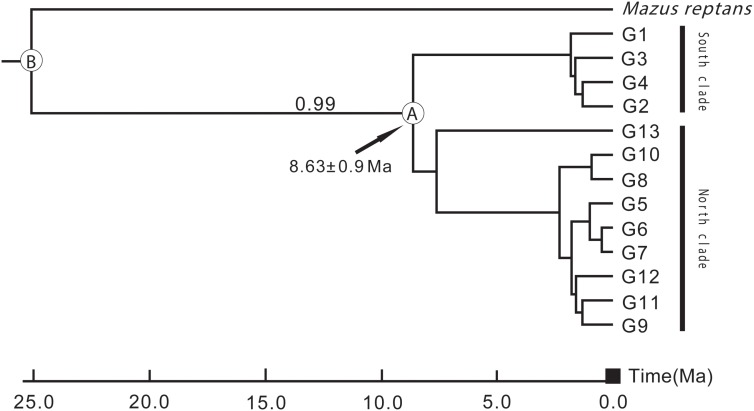
Divergence time between the major north and south lineages of *L. tibetica*, based on analysis of internal transcribed spacer regions. B indicates the divergence time of *L. tibetica* from *M. reptans*, and A indicates the divergence time between the north and south lineages.

According to preliminary calculations using ITS sequence data, *L. tibetica* diverged from *M. reptans* around 25 Ma, and the divergence of *L. tibetica* between the major north and south lineages was dated at around 8.63 Ma (Figure 6). These estimates of dates of origin need to be treated with caution but the estimated divergence times correspond well with the geological evidence of the QTP uplift during the late Miocene and Pliocene (Li and Fang, 1999; Zheng et al., 2000; Mulch and Chamberlain, 2006).

### Ecological Niche Modeling

**FIGURE 7:**
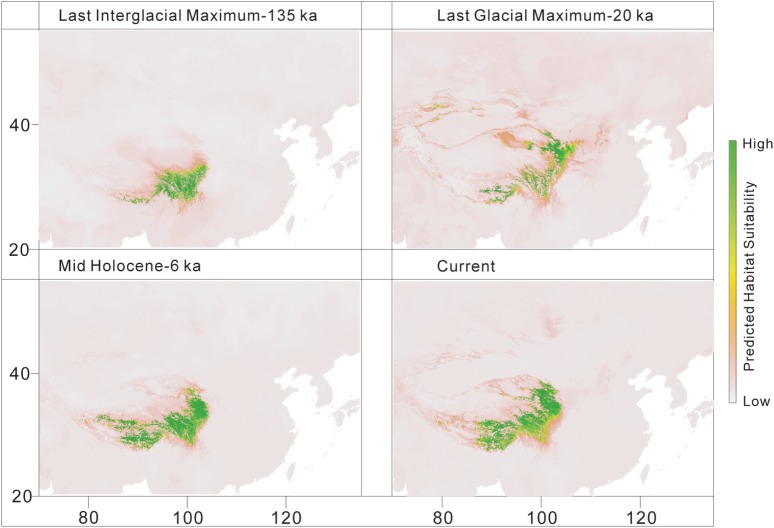
Distribution models for *L. tibetica*, simulated based on current, mid-Holocene (6 ka), last glacial maximum (20 ka), and last interglacial maximum (135 ka) periods.

**FIGURE 8:**
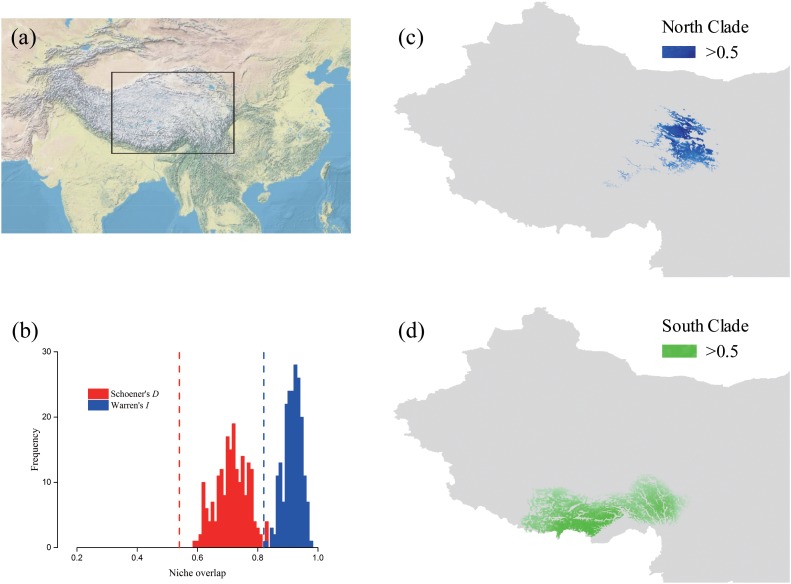
Potential distributions and niche overlap for *L. tibetica* lineages in the QTP. **(a)** The location illustration of the simulation area. **(b)** Vertical lines represent the empirical value of Warren’s *I* and Schoener’s *D* indices, obtained from observed points; the histograms represent the expected distribution of overlap; the null hypothesis of identical niches is rejected if the empirical value falls outside the 95% probability threshold of the expected distributions (*P* < 0.05). The potential distributions for *L. tibetica* in the North lineage **(c)** and South lineage **(d)**.

The predicted distribution of *L. tibetica* underwent significant changes during the glacial-interglacial period (Figure 7). From the last interglacial maximum to the last glacial maximum to the mid-Holocene, the range of the predicted distribution of *L. tibetica* experienced successive reduction and expansion. There was no significant change from the mid-Holocene to the current period (Figure 7). The AUC values for ecological niche modeling of the north and south lineages were 0.986 and 0.960, respectively, indicating far better than a random prediction. A test of identity between the two lineages showed that there was distinct niche differentiation (*P* < 0.05). A background test of both lineages also showed that the ecological niches of the two lineages are well differentiated (Figures 8a,c,d). Values of Schoener’s *D* and Warren’s *I* indices suggested significant niche divergence between the south and north lineages (*P* < 0.05, Figure 8b).

## Discussion

### Genetic Structure and Intraspecific Divergence

The average effective gene flow within the distribution range of *L. tibetica* is low, compared with that of previous studies on other species in the area, e.g., *Spiraea mongolica* (0.41) (Wang et al., 2014) and *Camellia flavida* (0.35) (Wei et al., 2017). Higher gene flow in those other species might have resulted in higher genetic differentiation among their populations. We found that the average effective gene flow among the two lineages of *L. tibetica* (0.149) was lower than that among the different populations (0.249). The seeds of *L. tibetica* are small and wingless and disperse near the parent plants, a feature that is likely to have enhanced the degree of genetic differentiation by restricting gene flow (Hong et al., 1998). However, we found high genetic differentiation among the populations, and most genetic variation was distributed among the populations and groups, based on SAMOVA. The geographic isolation of populations within species and variation in ecological factors are major driving forces to cryptic speciation (Hoskin et al., 2005; Liu et al., 2013).

The results of SAMOVA, the Bayesian inference tree and parsimony network analysis showed that *L. tibetica* comprises two major cpDNA groups. One group has its main geographic distribution to the north of the Tanggula and Bayangela Mountains, while the other group lies mainly to the south of the QTP. Gene flow across the whole region appears to be restricted by high mountains, suggesting a significant role of geography in the genetic differences between the two groups. Similarly, the ITS sequence variation showed clearly that the divergence of *L. tibetica* between the major north and south lineages was around 8.63 Ma. Although the estimates of dates of origin need to be treated with caution, they correspond well with geological evidence that the QTP experienced uplift during the late Miocene and Pliocene periods (Li and Fang, 1999; Zheng et al., 2000; Mulch and Chamberlain, 2006). This evidence suggests that the Tanggula and Bayangela Mountains appear to act as a geographical barrier for *L. tibetica*, probably imposed significant barriers on gene flow and divided the species into north and south lineages.

As indicated by the cpDNA, the ecological differences between the two lineages seem to represent species-specific characteristics that would be sufficient to keep the lineages separated to a high degree. Such distinct ecological niches would have reinforced the divergence of the two lineages following their initial spatial isolation. Thus, each of the two lineages may have given rise to some degree of differential adaptation to its respective environmental conditions. It is likely that the extensive QTP uplifts created fragmentation and isolation of habitats and niche differentiation, and provided the preconditions for the adaptive divergence of fragmented populations and subsequent speciation (Hewitt, 1996; Abbott and Brennan, 2014). In addition, about 9–8 Ma, enhanced aridity in the Asian interior and the onset of Indian and East Asian monsoons (Zhisheng et al., 2001) might have provided different ecological niches for the different lineages of *L. tibetica*. Some studies have reported that if related species live in significantly different niches, ecological divergence would likely be important in facilitating speciation, even in the presence of gene exchange (Nosil, 2008; Nosil et al., 2009a; Anacker and Strauss, 2014; Wan et al., 2016). Although ecological divergences in this case have not resulted in the emergence of new species, the initial divergence demonstrates the potential for ecological speciation. If geographic isolation and restricted gene flow are maintained long enough, they may eventually lead to reproductive isolation (Rieseberg and Burke, 2001; Nosil et al., 2009b; Thorpe et al., 2010), resulting in the formation of new species. Our results support the conclusion from previous studies that the uplift of the QTP and its associated climatic changes were most likely the main cause of plant diversification (Cun and Wang, 2010; Xu et al., 2010; Yang et al., 2012).

### Quaternary Demographic History and Glacial Refugia of *L. tibetica*

Climate changes during the Pleistocene glacial-interglacial cycles had a dramatic effect on species distribution ranges (Comes and Kadereit, 1998; Hewitt, 2004), causing migration and/or extinction of populations, followed by periods of isolation, divergence and subsequent expansion (Taberlet et al., 1998; Cun and Wang, 2010). During the Pleistocene period, continuing climatic oscillations caused repeated shifts in the abundance of alpine species (Tang and Shen, 1995; Herzschuh et al., 2010). There were some glacial refugia on the QTP platform, giving some plant species a chance to survive the changing climate (Yang et al., 2008; Wang et al., 2009, 2015; Li et al., 2011; Gao et al., 2016; Liu et al., 2018). In the present study, the simulated distribution from the last interglacial period to the current period showed that the distribution of *L. tibetica* experienced shrinkage and expansion (Figure 7). In the last glacial maximum period, extreme cold and dry weather substantially reduced its distribution from a continuous geographical distribution to a more fragmented pattern. Our distribution simulations for the mid-Holocene and the current period showed that the distribution ranges of *L. tibetica* were the same, and apparently more extensive than those in the last glacial maximum periods. Taken together, our results reveal that *L. tibetica* did experience population expansion.

Molecular data also provided further support for the above hypothesis. Based on cpDNA sequence variation, the north and south lineages of *L. tibetica* experienced a rapid range expansion at 0.172 Ma and 0.727 Ma, respectively (Table 5), consistent with the Pleistocene (Hewitt, 2000; Zheng et al., 2002). The largely open alpine regions that became available following the end of the major glaciation would have provided extensive opportunities for *L. tibetica* to expand its range. Indeed, such expansions of geographical range into alpine regions of the QTP have been reported previously for several plant species and are likely to have been common during the largest Pleistocene glaciation on the QTP (Liu et al., 2012, 2013; Gulzar et al., 2018; Lin et al., 2018). The QTP has been shown to be sensitive to climatic shifts, when plants were profoundly affected by alpine glaciation (Meng et al., 2007; Chen et al., 2008).

According to Taberlet and Cheddadi (2002), localities with high levels of genetic variation and unique haplotypes have often been recognized as possible refugia or as centers of diversification for species, whereas localities with low levels of genetic variation represent recent colonization. Some reports suggest that the mountainous areas of subtropical China may have provided refugia for warm-temperate evergreen species through periods of adverse climatic conditions (Bennett and Provan, 2008; Qiu et al., 2011; Wang et al., 2015). Our results showed that since the last glacial maximum, the south and north lineages experienced population expansion, while the population as a whole showed no expansion. In addition, even though *L. tibetica* has only two major lineages, some of its populations (9, 10, 12, 13, 14, and 15) contained higher haplotype and nucleotide diversities. It is likely that populations located in the Tanggula Mountains could survive in alternative habitats within a short distance, allowing biodiversity to persist during climate modifications (Hoorn et al., 2013). These patterns collectively suggest that areas south of the Tanggula and north of the Bayangela Mountains harbored refugia during the early Pleistocene, and then the southern and northern distribution ranges expanded rapidly at 0.727 and 0.172 Ma, respectively, during the interglacial periods. It might be a possible explanation for our finding that these populations have lower haplotype and nucleotide diversities than do some other species. Previous reports have suggested that rapid range expansion should decrease intra-population genetic diversity in the direction of spread (Hewitt, 1996; Soltis et al., 1997; Nason et al., 2002).

## Conclusion

In conclusion, analyses of *L. tibetica* from our sample range, bringing together molecular phylogeography and species distribution modeling, indicate that a combination of geographic isolation and climatic factors have played a fundamental role in promoting diversification and evolution of this species. This study provides valuable evidence that advances research on genetic differentiation on the QTP.

## Author Contributions

FZ and SC conceived and designed the experiments. MX and ZT performed the experiments. MX, ZT, FZ, and GK analyzed the data. QG, RX, YZ, and JY contributed reagents, materials, and analysis tools. MZ wrote the paper. FZ, SC, and GK reviewed and edited the paper. All authors approved the final version of the manuscript.

## Conflict of Interest Statement

The authors declare that the research was conducted in the absence of any commercial or financial relationships that could be construed as a potential conflict of interest.

## Footnotes

1 www.cvh.ac.cn
